# Live-cell fluorescence imaging of microgametogenesis in the human malaria parasite *Plasmodium falciparum*

**DOI:** 10.1371/journal.ppat.1010276

**Published:** 2022-02-07

**Authors:** Sabrina Yahiya, Sarah Jordan, Holly X. Smith, David C. A. Gaboriau, Mufuliat T. Famodimu, Farah A. Dahalan, Alisje Churchyard, George W. Ashdown, Jake Baum

**Affiliations:** 1 Department of Life Sciences, Imperial College London, London, United Kingdom; 2 Facility for Imaging by Light Microscopy, Imperial College London, London, United Kingdom; Loyola University Chicago, UNITED STATES

## Abstract

Formation of gametes in the malaria parasite occurs in the midgut of the mosquito and is critical to onward parasite transmission. Transformation of the male gametocyte into microgametes, called microgametogenesis, is an explosive cellular event and one of the fastest eukaryotic DNA replication events known. The transformation of one microgametocyte into eight flagellated microgametes requires reorganisation of the parasite cytoskeleton, replication of the 22.9 Mb genome, axoneme formation and host erythrocyte egress, all of which occur simultaneously in <20 minutes. Whilst high-resolution imaging has been a powerful tool for defining stages of microgametogenesis, it has largely been limited to fixed parasite samples, given the speed of the process and parasite photosensitivity. Here, we have developed a live-cell fluorescence imaging workflow that captures the entirety of microgametogenesis. Using the most virulent human malaria parasite, *Plasmodium falciparum*, our live-cell approach captured early microgametogenesis with three-dimensional imaging through time (4D imaging) and microgamete release with two-dimensional (2D) fluorescence microscopy. To minimise the phototoxic impact to parasites, acquisition was alternated between 4D fluorescence, brightfield and 2D fluorescence microscopy. Combining live-cell dyes specific for DNA, tubulin and the host erythrocyte membrane, 4D and 2D imaging together enables definition of the positioning of newly replicated and segregated DNA. This combined approach also shows the microtubular cytoskeleton, location of newly formed basal bodies, elongation of axonemes and morphological changes to the erythrocyte membrane, the latter including potential echinocytosis of the erythrocyte membrane prior to microgamete egress. Extending the utility of this approach, the phenotypic effects of known transmission-blocking inhibitors on microgametogenesis were confirmed. Additionally, the effects of bortezomib, an untested proteasomal inhibitor, revealed a clear block of DNA replication, full axoneme nucleation and elongation. Thus, as well as defining a framework for broadly investigating microgametogenesis, these data demonstrate the utility of using live imaging to validate potential targets for transmission-blocking antimalarial drug development.

## Author summary

Upon transmission of *Plasmodium*, the causative agents of malaria, male and female gametes form in the mosquito midgut in a remarkably rapid and complex cellular transformation. Microgametogenesis, or male gamete formation, describes the formation of eight haploid microgametes from a single gametocyte in only 15 minutes. Simultaneous egress from the host erythrocyte, substantial cytoskeletal rearrangement and DNA replication occurs during this characteristically explosive transformation. Whilst microgametogenesis has been extensively studied using various microscopy techniques, efforts to date have been limited by the need for complex transgenic parasite generation and fixed-parasite protocols. Here, we have developed a live-cell fluorescence imaging approach to study microgametogenesis, overcoming both the laborious staining steps and spatiotemporal limitations of existing approaches. Crucially, our newly devised workflow utilises widefield fluorescence microscopy, commercially available stains and an open-access analysis software and is hence accessible to the wider malaria research community. We demonstrate the ability to retrieve and quantify volumetric data from our live-cell approach and highlight the applicability of our live-cell approach to transmission-blocking drug discovery. Using our workflow, we have elucidated the cellular phenotypes of both existing and unknown inhibitors of microgametogenesis. Using this approach, we find the proteasome to be a potential microgametogenesis-blocking cellular target.

## Introduction

Malaria disease is caused by single-cell protozoan parasites from the genus *Plasmodium*. Over its complex two-host lifecycle, the *Plasmodium* cell demonstrates remarkable cellular plasticity as it transitions between multiple developmental stages. In the transition from mammalian to mosquito host, the parasite faces an extreme population bottleneck in numbers, which also presents a natural target for novel antimalarial treatments aimed at blocking transmission. Transmission is triggered by the uptake of sexual stage gametocytes during a mosquito feed that instantly activate, initiating a transformation in the mosquito midgut that has become a trademark in the cellular biology of these protozoan parasites [1].

Dormant male (micro) and female (macro) gametocytes form a sub-population of between 0.2–1% of the circulating asexual blood stage parasite reservoir in the mammalian host. The signals that initiate commitment of asexual parasites to sexual differentiation are, however, poorly understood [1]. Committed gametocytes mature over five distinct morphological stages (referred to as stages I-V) and are believed to sequestrate in the host bone marrow and spleen, before emerging into the bloodstream when they reach stage V maturity [1–3]. Following ingestion by a feeding mosquito, stage V microgametocytes and macrogametocytes transform rapidly to microgametes and macrogametes, respectively. The transformation from gametocyte to gamete, a process termed gametogenesis, is activated by a decrease in temperature to 20–25°C, rise in pH and the presence of the mosquito metabolite, xanthurenic acid in the mosquito midgut [4].

*Plasmodium* gametogenesis is distinctly different between male and female parasites. In the most virulent human malaria parasite, *Plasmodium falciparum*, gametogenesis commences with a morphological change from falciform to rounded that precedes egress from within the host erythrocyte by an ‘inside-out’ mechanism, the latter found in all *Plasmodium* spp. This mechanism of egress involves disintegration of the parasitophorous vacuole membrane (PVM) prior to that of the host erythrocyte [5]. The female macrogametocyte rounds up [6] and egresses [5] within 10 minutes of activation, emerging as a fertilisation competent macrogamete. Whilst this process is incompletely understood, reverse genetic studies using different *Plasmodium* species have described some key female specific events underlying macrogametogenesis [7–11], such as release of osmiophillic bodies—membrane-bound organelles which are sparse if not entirely absent in microgametocytes [12]. Whilst females become fertilisation-competent upon egress and undergo little cellular reorganisation beyond rounding, microgametogenesis is notoriously complex, and is the focus of our study here.

Microgametogenesis has been most extensively studied by electron microscopy (EM) investigation of the rodent malaria parasite, *P*. *berghei* and *P*. *yoelii* [13–15]. The detailed EM work has revealed that microgamete formation entails a stepwise series of events including: substantial cytoskeletal rearrangement, three rounds of DNA replication, alternating with three rounds of endomitotic division, all of which occurs in ~15–20 minutes. The process begins with the characteristic falciform microgametocyte, the form that gives the species its name [2, 3], morphologically transforming to round once activated. Upon activation, a single microtubule organising centre (MTOC) develops into two orthogonal tetrads of basal bodies attached to a spindle pole. The resulting eight basal bodies, from which eight axonemes nucleate and elongate, segregate with each endomitotic division [14, 16, 17]. Axoneme assembly occurs, fuelled by the large quantities of tubulin within mature microgametocyte that rapidly polymerises to form microtubules [18]. Prior to activation, the MTOC is positioned in close alignment with the nuclear pore, permitting each basal body to pull a haploid genome (1n) from the newly replicated octoploid (8n) genome through the parental cell body at the point of emergence [14]. This dynamic process by which developing haploid microgametes emerge as motile flagellum is termed exflagellation and occurs from ~15 minutes post-activation [16]. Motile haploid microgametes then fuse with the sessile macrogamete, producing a motile zygote able to migrate to the midgut epithelium for oocyst formation and onward progression in the mosquito [19].

Low and high-resolution light microscopy of *Plasmodium* has been widely used to explore parasite cell development and aid drug-intervention studies, predominantly using fixed fluorescence microscopy [20–22]. This has been extended to high throughput screening, combining drug discovery with automated fluorescence imaging. Use of high throughput imaging has helped screen for phenotypes of transmission blocking or pre-erythrocytic targeting antimalarial hits with unknown cellular targets [23, 24]. More recently, other approaches like ultrastructure expansion microscopy have advanced traditional fluorescence approaches to allow more detailed observations of asexual blood stage, microgametocyte and ookinete cytoskeletal development [25]. Low-resolution, live brightfield imaging of exflagellation in a high-throughput assay [26] has been used to identify microgametogenesis-blocking hits [23], whilst other studies have reported use of live-cell fluorescence imaging of microgametogenesis to define the phenotypes of transgenic *P*. *berghei* [27, 28] cell lines. The highest resolution live imaging to date has used custom built platforms based on either Stimulated Emission Depletion (STED) microscopy [29] or lattice light-sheet microscopy [30], both of which have proven to be valuable in the study of *P*. *falciparum* asexual blood stage development. The cost and requirement on bespoke equipment, however, makes such approaches inaccessible to most research groups.

Application of live or ultrastructural imaging to microgametogenesis has been limited. Microtubular dynamics, which play a key role in the process [12] are not easily resolvable by brightfield and the specificity of fluorescent imaging often requires antibody labelling, limiting imaging to fixed samples. This is also true for electron microscopy, despite its key role in shaping our current understanding of the fine cellular biology of microgametogenesis [13, 14, 31]. As a result, the dynamic nature of events encompassing this critical dynamic event in parasite transmission are still very poorly understood. Better temporal characterisation using live samples, coupled with the specificity of fluorescently tagged structures would allow a marked improvement in our understanding of the process of microgametogenesis and provide a platform from which strategies to block it might then be translatable.

Here, we describe a protocol that captures the entire process of microgametogenesis in *P*. *falciparum* microgametocytes, utilising specific labels for microtubules, DNA and the host erythrocyte membrane and imaging using an approach that alternates between live-cell 3D fluorescence microscopy through time (4D imaging), brightfield imaging and 2D fluorescence microscopy. To develop a workflow that is translatable to other research labs, we have used a combination of widefield microscopy, an open-source analysis software for deconvolution and commercially available reagents throughout this study. Using this approach, we define in detail the dynamic morphological transformations that occur across the entirety of microgametogenesis, from activation through to exflagellation. Furthermore, we demonstrate the applicability of our imaging approach to phenotypic characterisation of inhibitors of microgametogenesis, in particular those that block the proteasome, demonstrating the power of live microscopy in future transmission-blocking drug discovery [20].

## Results

### Development of a live microgametogenesis imaging approach

**Fig 1:**
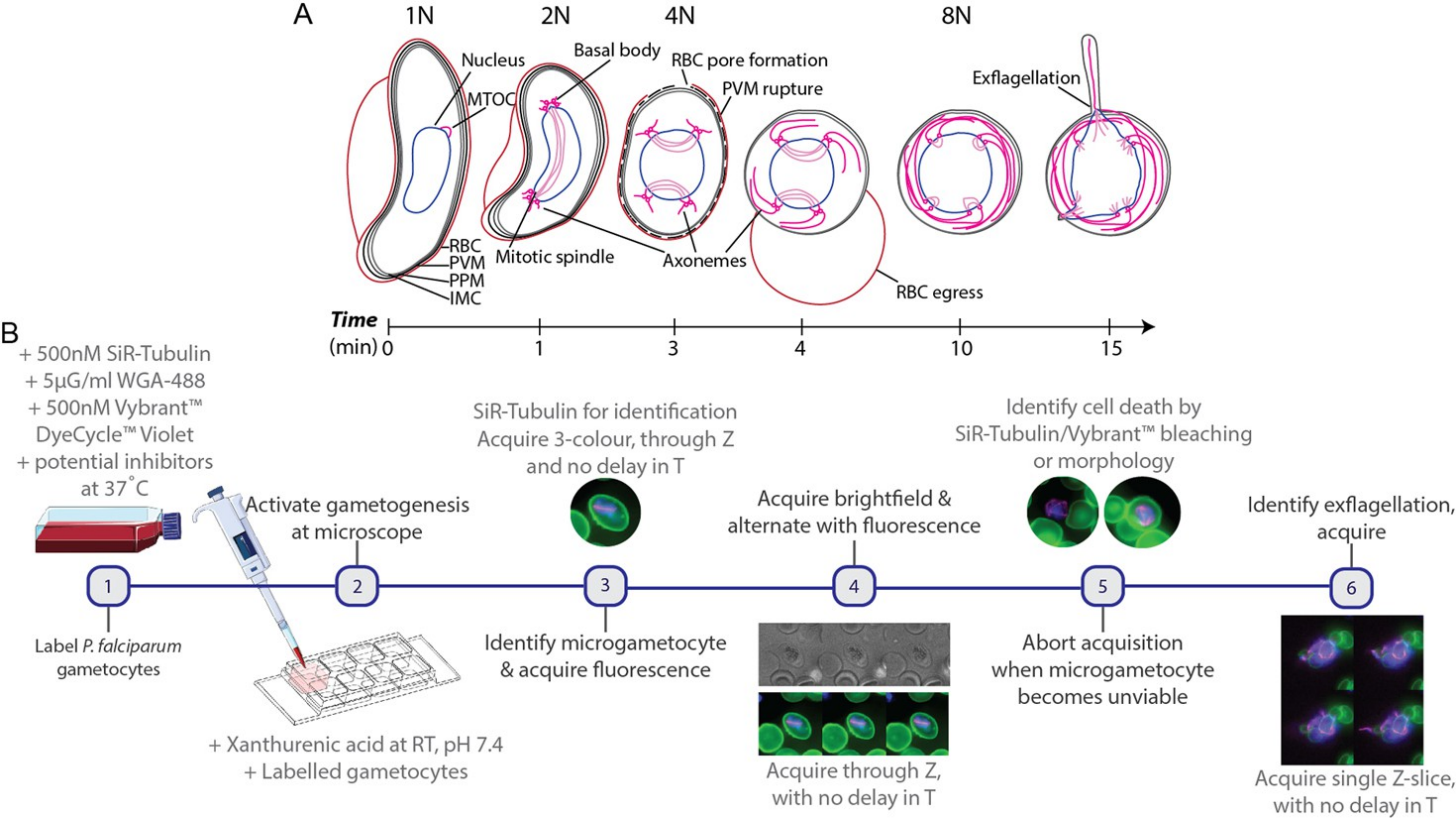
*P*. *falciparum* microgametogenesis and our live cell imaging approach. **(A)** Details of the cell biological transformations occurring during microgametogenesis from activation at t = 0 minutes to exflagellation at t = 15 minutes. At t = 0 minutes, microgametocytes start with a falciform morphology and an in-tact 4-layer membrane, comprised of the red blood cell (RBC) membrane, parasitophorous vacuole membrane (PVM), plasma membrane (PPM) and inner membrane complex (IMC). Following the first round of DNA replication (1n-2n) at t = 1 minute, the microtubule organising centre (MTOC) transforms to two tetrads of basal bodies joined by a mitotic spindle. Further replication of DNA (2n-4n, 4n-8n) occurs after ~ t = 3–4 minutes, simultaneously to the separation of basal bodies and egress. During egress, PVM rupture precedes erythrocyte egress (inside-out-mechanism) and parasites egress from an erythrocyte pore. Axonemes nucleate from basal bodies following the first DNA replication at t = 1 min and elongate from t = 1–15 min, coiling around the parasite cell body. At 15 minutes post-activation, axonemes emerge attached to a haploid genome as microgametes, in the process of exflagellation. **(B)** The workflow of live gametocyte labelling and fluorescence microscopy of microgametogenesis. *P*. *falciparum* NF54 gametocytes are labelled with SiR-Tubulin, WGA-488 and Vybrant DyeCycle Violet at 37°C, at which point inhibitors of microgametogenesis may be added. Labelled gametocytes are subsequently activated with ookinete medium in pre-positioned imaging slides at room temperature (RT). SiR-tubulin labelled mitotic spindles are used to identify microgametogenesis events, which are continually imaged through T and Z, alternating between brightfield and fluorescence to minimise phototoxicity. When parasites are deemed unviable based on photobleaching or morphology, exflagellation events are captured as single Z-slice timelapses through T. Panel B was created using Servier Medical Art templates, which are licensed under a Creative Commons Attribution 3.0 Unported License; https://smart.servier.com.

To date, visualisation of the complex cytoskeletal rearrangement, host erythrocyte egress and DNA replication events during *P*. *falciparum* microgametogenesis (**Fig 1A**) has mostly been limited to fixed imaging protocols. We set out to devise a live cell imaging workflow (**Fig 1B**) that permits observation of cellular dynamics during microgametogenesis in real-time and in three dimensions. Selective testing of several DNA dyes revealed that 4′,6-diamidino-2-phenylindole (DAPI) and silicon-rhodamine (SiR) derivative SiR-DNA failed to effectively label live *P*. *falciparum* gametocytes. With optimisation, however, Vybrant DyeCycle Violet in combination with the silicon-rhodamine (SiR) derivative SiR-tubulin, and wheat germ agglutinin (WGA) could effectively label live microgametocyte DNA, microtubules and the host erythrocyte membrane, respectively. Vybrant DyeCycle Violet is a cell permeable dye which binds to double-stranded DNA to emit a fluorescent signal proportional to DNA mass and has been previously used to measure microgametocyte genome replication during microgametogenesis [32, 33] using flow cytometry. SiR-tubulin, a non-toxic far-red fluorogenic probe [34], is an SiR-derivative conjugated to docetaxel [35] which binds specifically to microtubules.

Stage V gametocytes from the *P*. *falciparum* NF54 strain, were cultured as previously described [36], incubated with dyes and strictly maintained at 37°C to prevent premature activation of gametogenesis. Gametogenesis was initiated by mimicking conditions of the mosquito midgut using “ookinete media” (see **Materials and Methods**), a xanthurenic-acid-containing media maintained at pH 7.4 and used at room temperature (RT). Labelled gametocytes were directly added to ookinete media-containing imaging slides and prepositioned on the microscope for the immediate acquisition of time-lapse data (**Fig 1B**).

To visualise the initial developmental stages of microgametogenesis, microgametocytes were identified by SiR-tubulin-labelled mitotic spindles which signified a successful round of DNA replication (**Fig 1A**). Due to the rapid turnaround between activation and DNA replication, most time-lapses presented here were acquired from 1–2 minutes post-activation. It has been previously reported that the first round of DNA replication is initiated within 20 seconds of activation [32], and hence acquisition of time-lapses here followed a round of replication. In optimisation of the imaging workflow, we found the alternation between fluorescence and brightfield acquisition to significantly maximise the viability of microgametocytes. Following identification of an activated microgametocyte, 3-colour fluorescent time-lapses were immediately acquired before switching to brightfield microscopy. Fluorescent time-lapses were acquired as a maximum of 10 frames per time-lapse, before switching to brightfield microscopy to both monitor parasite development and minimise phototoxic effects of fluorescence microscopy. Upon observation of further parasite differentiation in brightfield, for example by rounding up or preparing for egress, image acquisition was switched back to fluorescence for further acquisition of 10-frame time-lapses. Fluorescent time-lapses were acquired every 1–3 minutes to capture the full early developmental stages of microgametogenesis, acquiring approximately 24–44 Z-slices to encompass the full parasite body.

Despite best efforts to reduce LED intensity and frame rates, phototoxicity nearly always prevented the complete visualisation of microgametogenesis from start to finish. To circumvent this issue, activated microgametocytes were imaged at different stages to ensure full capture of later developmental stages, specifically the emergence of microgametes during exflagellation (**Fig 1A**). In this late-stage instance, viable microgametocytes were identified based on SiR-tubulin-labelled axonemes, coiled around the parasite cell body. Whilst the earlier stages of microgametogenesis were acquired in 4D, through Z and T, exflagellation could only be captured as single Z-slice time-lapses (2D fluorescence imaging) given the dynamic and unpredictable nature of emerging microgametes (**Fig 1B**). Time-lapse data of the early and later stages of microgametogenesis were subsequently combined and could be analysed together, enabling us to dissect microgametogenesis in its entirety, from initial endomitotic division through to microgamete emergence, for the first time.

### Insights into cytoskeletal rearrangements during microgametogenesis

The formation of mitotic spindles, basal bodies and axonemes occurs with rapid succession during the early stages of microgametogenesis [14, 15]. Using our approach and the advantages of alternating between brightfield, 2D fluorescence and 3D microscopy we sought to define these stages in real time.

**Fig 2:**
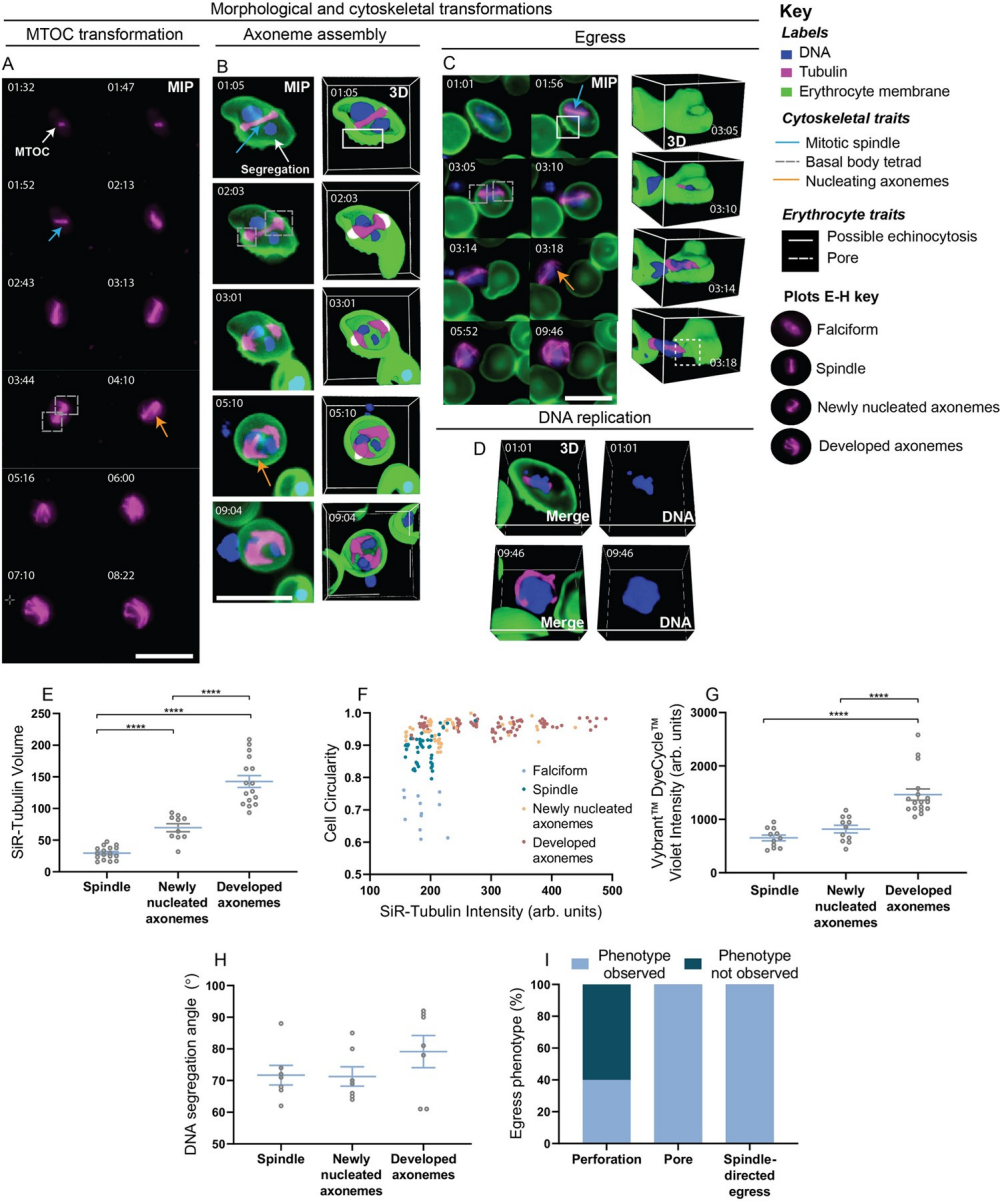
Tubulin dynamics, egress and DNA replication during *P*. *falciparum* microgametogenesis. Individual time-frames acquired from fluorescent time-lapses of microgametocytes labelled with **(A)** SiR-Tubulin (magenta) only and **(B-D)** a combination of SiR-Tubulin (magenta), WGA-488 (green) and Vybrant DyeCycle Violet (blue) in the early developmental stages of microgametogenesis. See **S1**, **2** and **3** Videos for corresponding timelapses of **A**-**C**. **(A)** Microtubule labelling (magenta) portrays MTOC transformation into basal body tetrads and axoneme nucleation, depicted as maximum intensity projections (MIP) of 3D data. **(B-D)** DNA replication (blue), microtubule dynamics (magenta), host erythrocyte egress and morphological transformations (host erythrocyte membrane, green). **(B)** Microtubule labelling showing the formation of mitotic spindles, basal bodies and axonemes. DNA (blue) segregation is visible. **(C)** Erythrocyte membrane possible echinocytosis (white dashed box), pore formation (white dashed box) and egress are shown. **(D)** A change in DNA content between early and late stages is visible. **(E)** SiR-Tubulin volume of individual cells from distinct developmental stages; spindle (*n* = 17), newly nucleated axonemes (*n* = 10) and developed axonemes (*n* = 14). **(F)** A graph plotted to show cell circularity and SiR-Tubulin intensity (arbitrary units), with each plot representing individual cells of a given developmental stage; falciform (*n* = 12), spindle (*n* = 46), newly nucleated axonemes (*n* = 48) and developed axonemes (*n* = 82). Representative images of developmental stages are above the plots. **(G)** Vybrant DyeCycle Violet intensity (arbitrary units) quantified in distinct developmental stages; spindle (*n* = 11), newly nucleated axonemes (*n* = 11) and developed axonemes (*n* = 17). **(H)** DNA segregation angles measured between basal body tetrads and segregated DNA are shown (*n* = 7). **(I)** Egress phenotypes were quantified (*n* = 5). Significant differences in label intensities between developmental stages were calculated using one-way ANOVA tests with Tukey multiple comparisons (**** *p* < .0001). **A-D** Time is depicted as minutes and seconds (mm:ss). 2D maximum intensity projection (MIP) of 3D data, scale bars = 10 μm. **B-D** 3D projections of volume rendered data are shown. Individual channels of **B** and **C** can be found in **S1A** and **S1B Fig**, respectively. **A-D** All images here depict the observations made from >10 biological replicates.

Consistent with existing knowledge of microgametogenesis, mitotic spindle formation started out as a single MTOC that then transformed into two tetrads of basal bodies upon the first round of DNA replication (**Fig 2A** and **S1 Video**). As depicted in **Fig 2B**, the mitotic spindle of a developing microgametocyte first formed and lengthened across the width of the parasite. Although the ultrastructure of basal bodies was not as finely resolved as it is with conventional electron microscopy techniques, microtubule labelling nonetheless revealed basal body tetrads to form at the extreme ends of mitotic spindles. Axonemes were then seen to nucleate and elongate from each basal body, coiling around the parasite, as shown in **Fig 2B** and **S2 Video**. As evident in the 3D data (**Fig 2B** and **S2 Video**), SiR-tubulin labelling gathered at spindle poles whilst four axonemes were nucleated and elongated from basal bodies. We quantified the SiR-tubulin labelling volume of microgametocytes for three distinct developmental stages: microgametocytes with a fully formed spindle; newly nucleated axonemes; and developed axonemes (**Fig 2E**). A significant increase in SiR-tubulin volume was quantified across the three stages (**Fig 2E**), representing the rapid transformation of soluble tubulin into microtubules that occurs during microgametogenesis. This finding was consistent with previous EM studies of microgametogenesis [14, 15]. Notably, we have demonstrated the ability to retrieve and quantify volumetric data when 4D imaging data is captured, which, prior to this study, has not been possible by EM and limited to fixed samples in immunofluorescence studies.

Simultaneous with cytoskeletal rearrangements, microgametocyte morphology was seen to transform from falciform to round, changing the morphology of the host erythrocyte in the same way (**Fig 2B and 2C** and **S2 and S3 Videos**). To identify the relationship between rounding up and microtubule polymerisation, we quantified microgametocyte cell circularity and SiR-tubulin intensity, respectively. Individual cells were characterised across developmental stages: microgametocytes that were falciform or had a fully developed mitotic spindle, versus those with newly nucleated axonemes or developed axonemes. We found that falciform and spindle-containing parasites at the initial stages of microgametogenesis showed a similarly low level of SiR-tubulin intensity, but varying levels of circularity as they transformed from falciform to round. In contrast, parasites with newly nucleated or developed axonemes reached maximal circularity and showed the greatest increase in SiR-tubulin intensity. This relationship between circularity and SiR-tubulin intensity suggests that most microtubule polymerisation occurs in fully rounded parasites during axoneme elongation, whereas the initial stages of rounding and mitotic spindle formation require markedly less microtubule polymerisation (**Fig 2F**).

### Microgametocyte DNA segregates and localises perpendicularly to spindle poles

Incorporating the DNA dye, Vybrant DyeCycle Violet, to label microgametocytes, we next explored the behaviour of the nucleus. Using 3D sectioned data derived from our 4D fluorescence dataset, we observed nuclear segregation to occur as the microgametocyte genome was replicated (**Fig 2B** and **S2 Video**). When comparing the localisation of tubulin and DNA labelling, we noted that segregated DNA positioned perpendicularly to basal body tetrads (**Fig 2B** and **2H** and **S2 Video**). To quantify this novel observation, angles between segregated DNA and basal body tetrads were measured under three distinct developmental stages: microgametocytes displaying either a i) mitotic spindle, ii) newly nucleated axonemes or iii) developed axonemes. Using volumetric data, acquired through time to enable the selection of appropriate time-frames and optimal orientation of images required to measure the angle of segregation, segregated DNA labelling and basal body tetrads were found to consistently angle between 60 and 90° in each of the stages (**Fig 2H**). This supports the observation that nuclear segregation occurs at opposing angles.

Upon full axoneme development, DNA content visibly increased compared to earlier stages of microgametogenesis (**Fig 2D**). When quantified, a significant increase of DNA labelling from spindle formation to nucleation and development of axonemes was found (**Fig 2G**). These data demonstrate that volumetric measurements can be obtained using our imaging framework, permitting real-time quantification of DNA content and providing a unique window into genome replication and the spatial localisation of events within the dividing microgametocyte.

### Host erythrocyte pore formation and possible echinocytosis in preparation for microgametocyte egress

A key stage in microgametogenesis is parasite egress from the host erythrocyte (**Fig 2C** and **S3 Video**). Live imaging of microgametogenesis revealed that the erythrocyte membrane likely undergoes echinocytosis in preparation for egress, as evident from the formation of small, spiked projections on the cell surface (**Fig 2C**). Although echinocytosis is an established feature of asexual blood stage development post-invasion [37], the phenomenon is reported here as a feature of microgametocyte-egress, for the first time. Activated microgametocytes aligned at the periphery of the parasite membrane and eventually ejected from a frequently singular pore that formed in the host erythrocyte (**Fig 2C** and **S3 Video**). In contrast to the projections visible on the surface of the host erythrocyte due to possible echinocytosis, a single opening was observed at the erythrocyte during pore formation. Notably, we observed a single spindle pole of the parasite to align towards to host erythrocyte pore, before ejecting in this direction (spindle-directed egress) (**Fig 2C** and **S3 Video**). Quantification of these observations revealed that, whilst possible echinocytosis was not the most abundant phenotype, pore formation and ejection from the spindle pole (spindle-directed egress) occurred in each instance of egress (**Fig 2I**). This finding suggests the mechanism of microgametocyte egress may utilise an unexplored driving force that coordinates ejection from the host cell with spindle pole positioning.

### Real-time fluorescence imaging of exflagellation

The final steps of microgametogenesis are the emergence of haploid microgametes from the parasite cell body, the remarkably dynamic process of exflagellation. Upon full elongation, the tip of an axoneme aligns at the periphery of the parasite cell body to emerge. When emerging at ~15 minutes post-activation, the developed axoneme carries a haploid genome (1n) from the newly replicated octoploid genome (8n) through the cell surface (**Fig 1A**).

**Fig 3:**
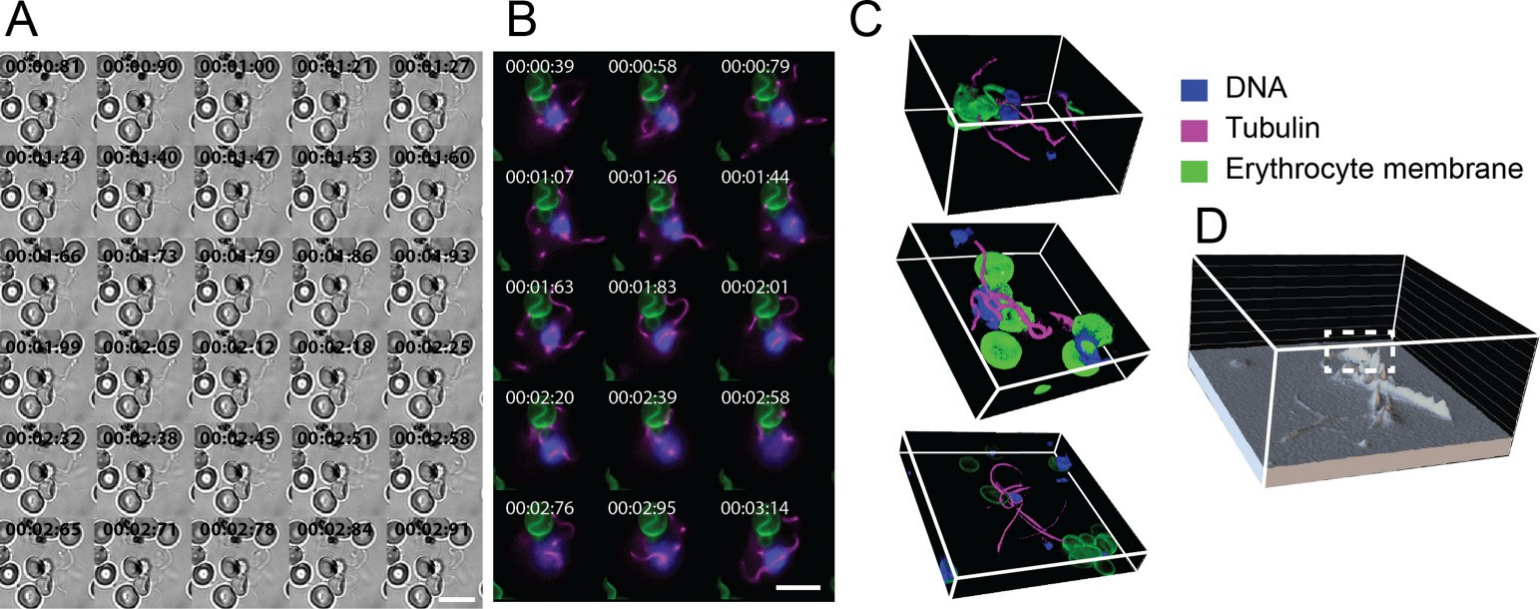
Exflagellation of *P*. *falciparum* microgametes. 2D individual time-frames acquired from timelapses of exflagellation imaged by **(A)** brightfield and **(B-C)** fluorescence microscopy. See S8 and S15 **Videos** for corresponding timelapses of **A**-**B**. **(B-C)** SiR-Tubulin-labelled axonemes (magenta) emerge from the parasite cell as microgametes. **(B)** Emerging microgametes carry a 1n genome from the newly replicated 8n genome (blue) and adhere to neighbouring erythrocytes (green). **(C)** 3D projections of volume rendered data of exflagellating microgametes, see **S17 Video** for a 3D rotated view. **(D)** 3D intensity plot of SiR-Tubulin labelling intensity to reveal dense regions (white dashed line) of axoneme overlap. **A-C** Time is depicted as minutes, seconds and milliseconds (mm:ss:ms). Scale bars = 10 μm. Individual channels of **C** can be found in **S1E Fig**. All data depicted reflect observations from >10 biological replicates.

Emerging microgametes could be identified either by SiR-tubulin labelled axonemes or by the increased motion visible in brightfield, with the latter minimising the effects of phototoxicity. Following the initial emergence of microgametes (**S2 Fig** and **S7 Video**), the full length of axonemes continued to emerge in a rapid motion (**Fig 3**). Full microgamete lengths were visualised by brightfield (**Fig 3A** and S8 **and S9 Videos**) and SiR-tubulin (**Fig 3B** and **S10–S17 Videos**). Combined observations of multiple developed axonemes and the high DNA content of exflagellating parasites indicated the formation of a replicated, likely octoploid, genome. This points to the preparation for multiple axonemes to emerge along with a genome, as haploid microgametes.

Due to the dynamic nature of emerging microgametes that were motile through Z-acquisition, exflagellation was captured as single-frames (2D) rather than 3D frames through time (4D imaging). Although 3D frames of exflagellation were not obtained and volumetric data was thus not captured at these stages, we instead measured regions of dense-labelling, for example by obtaining plots of SiR-tubulin intensity labelling from 2D datasets (**Fig 3D**). Using the example of SiR-tubulin, this allowed the identification of dense regions of labelled microtubules resulting from stacked axonemes yet to emerge by exflagellation using our 2D imaging data (**Fig 3D**). This technique therefore enabled us to make inferences on the 3D alignment of parasites without volumetric data. Alternatively, as parasite motion halts during loss of viability, 3D data can be obtained immediately after, to closely observe the positioning of emerged microgametes (**Fig 3C** and **S17 Video**), demonstrating our ability to acquire both 2D and 3D exflagellation data.

### Drug inhibition of microgametogenesis

**Fig 4:**
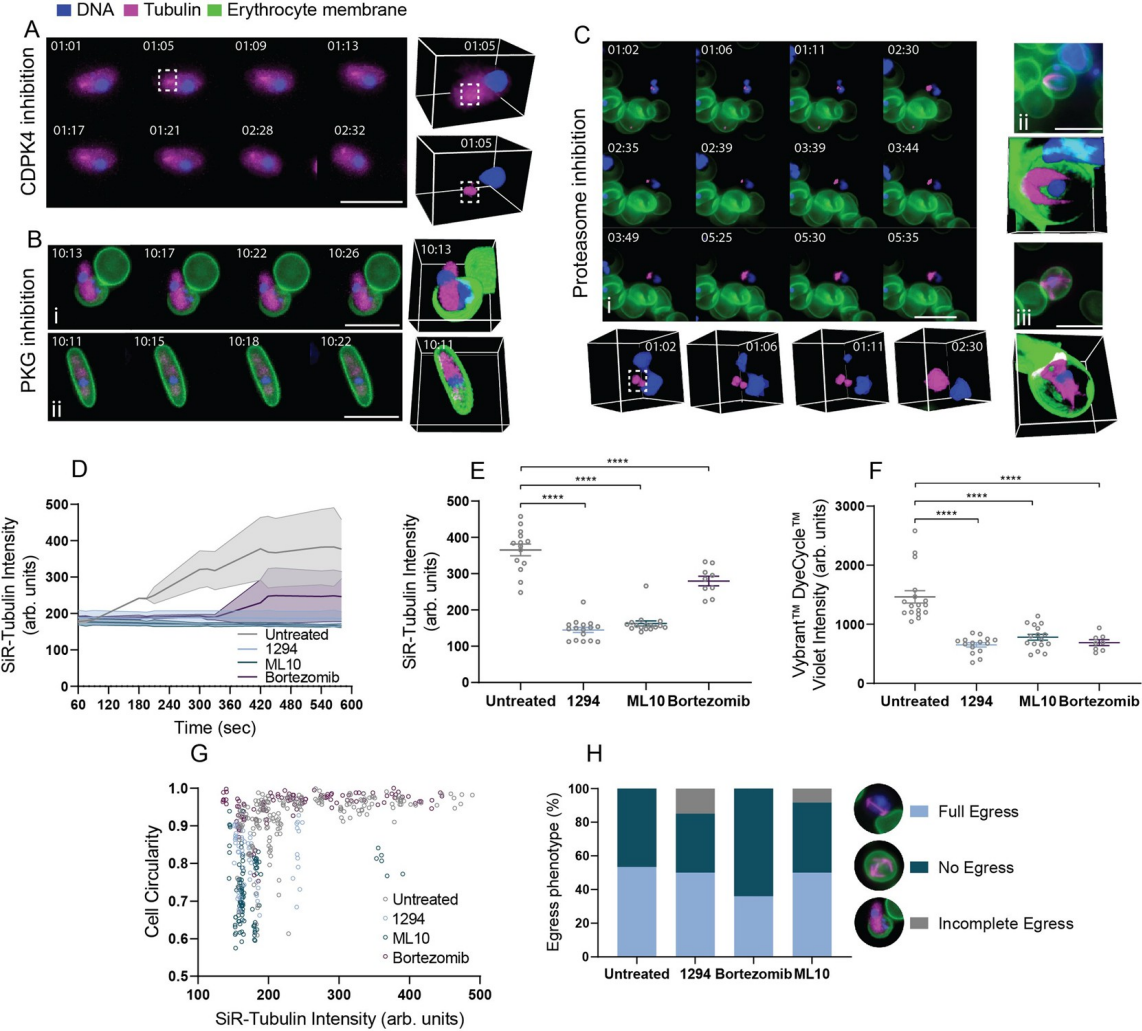
Cellular phenotypes of PKG, CDPK4 and proteasome-inhibited parasites. Cellular phenotypes upon inhibition of *P*. *falciparum* **(A)** CDPK4, **(B)** PKG and **(C)** proteasome by 1294, ML10 and bortezomib, respectively, during microgametogenesis. Perturbations to microtubule rearrangement (SiR-Tubulin, magenta), the host erythrocyte (WGA, green) and DNA replication (Vybrant DyeCycle Violet, blue) are shown as 2D maximum intensity projections of 3D data and alongside 3D sectioned views. Individual channels can be found in **S3 Fig**. **(A)** Failed DNA replication, cytoskeletal rearrangement and MTOC (white dashed line) transformation under 1294-treatment are shown. Stress induced egress prior to activation is also depicted. See **S19 Video**for the corresponding time-lapse. **(B)** The failed DNA replication and cytoskeletal rearrangement due to PKG inhibition by ML10 is shown. Mixed egress phenotypes were observed, including **(i)** incomplete and **(ii)** failed egress. See corresponding timelapses in **S23 and S24 Videos**. **(C)** Perturbations to **(i)** MTOC transformation (white dashed line) and **(ii-iii)** formation of two-three truncated axonemes resulting from proteasome inhibition are shown. See S25, S27 and S28 **Videos** for the corresponding timelapses. **(D)** A continuum of SiR-tubulin labelling intensity (arbitrary units) in untreated (*n* = 4), 1294 (*n* = 3), ML10 (*n* = 3) and bortezomib (*n* = 6) treated parasites. **(E)** SiR-tubulin labelling (arbitrary units) at 10 minutes post-activation under different treatments. Untreated (*n* = 14), 1294 (*n* = 16), ML10 (*n* = 16), bortezomib (*n* = 9). Significance was calculated using one-way ANOVA tests with Tukey multiple comparisons (**** *p* < .0001). **(F)** Vybrant DyeCycle Violet labelling (arbitrary units) was significantly reduced (one-way ANOVA tests with Tukey multiple comparisons (**** *p* < .0001)) at 10 minutes post-activation under different treatments. Untreated (*n* = 17), 1294 (*n* = 15), ML10 (*n* = 9), bortezomib (*n* = 16). **(G)** A graph depicting the cell circularity and SiR-tubulin labelling intensity of individual cells across the entirety of microgametogenesis under varying treatments. Untreated (*n* = 188), 1294 (*n* = 58), ML10 (*n* = 106), bortezomib (*n* = 105). **(H)** Percentage egress at 10 minutes post-activation under different treatments was quantified, with distinct egress phenotypes depicted beside the stacked bar graph. Untreated (*n* = 58), 1294 (*n* = 20), ML10 (*n* = 25), bortezomib (*n* = 24). All imaging data depicted reflect observations from >3 biological replicates.

The process of microgametogenesis is tightly synchronised by a series of cell cycle regulators and is consequently sensitive to, and the target of, known and developmental drug treatments [23]. We sought to apply our imaging approach as a drug discovery tool that could help elucidate the cellular phenotypes of compounds with known and unknown activity against microgametogenesis regulators towards defining their mode or process of action. Compounds 1294 [32, 38] and ML10 [39] have been well-established as potent inhibitors of microgametogenesis regulators Ca^2+^-dependent protein kinase 4 (CDPK4) and cyclic-GMP dependent protein kinase G (PKG), respectively. CDPK4 tightly regulates three processes during microgametogenesis: initiation of the first genome replication, mitotic spindle assembly and microgamete motility [32]. PKG has roles in the regulation of Ca^2+^ levels and rounding up during gametogenesis [40, 41]. The cellular phenotype of 1294 [32, 38] and ML10 [39] have been previously reported using immunofluorescence labelling of fixed microgametocytes [42]. Using our imaging approach, we could resolve distinct cellular phenotypes for each drug (**Fig 4** and **S18–S24 Videos**).

**Fig 5:**
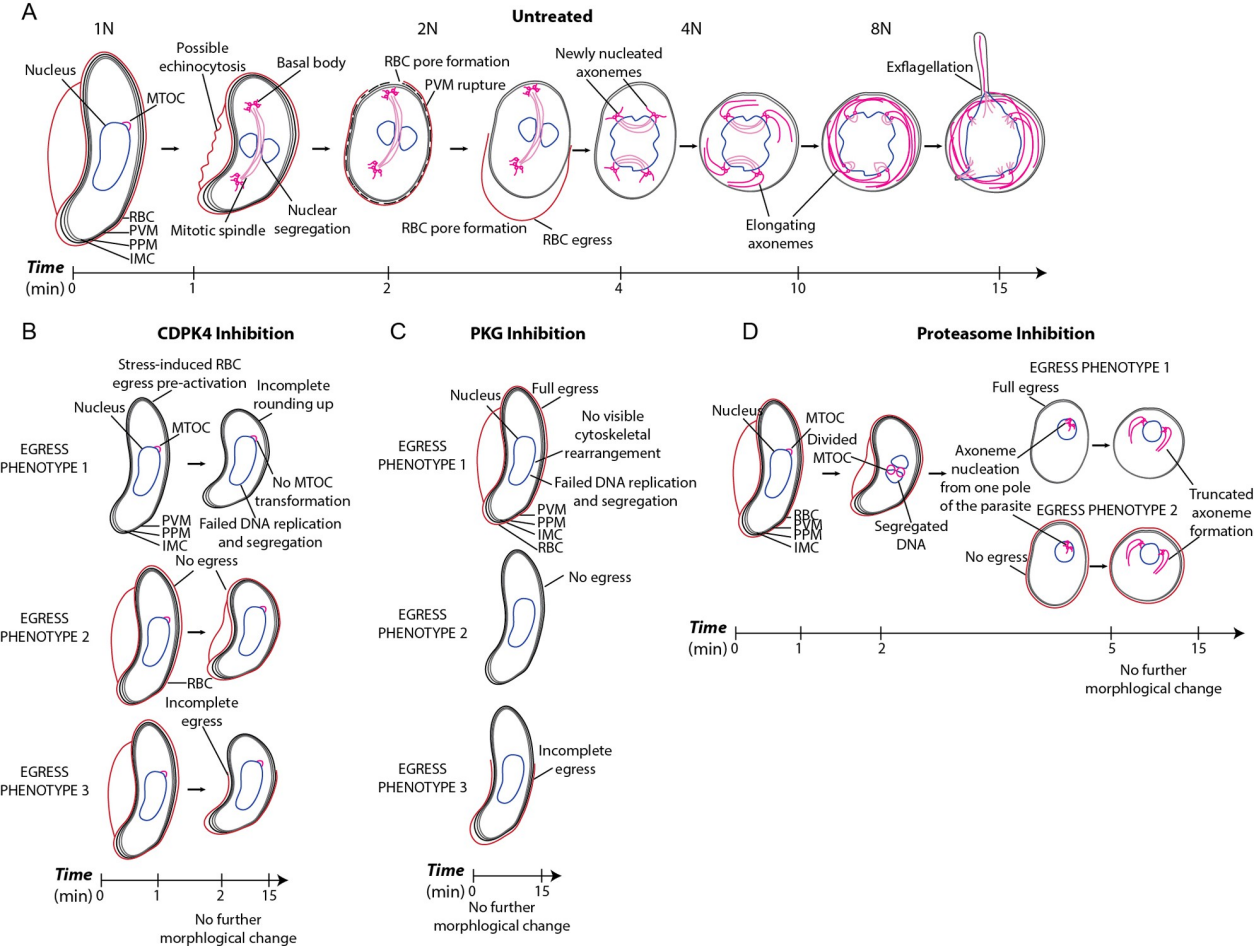
Novel insights into microgametogenesis with and without drug inhibition. Schematic diagrams showing the transformations observed by our live-cell microscopy of microgametogenesis. The observations depicted are of **(A)** untreated, **(B)** CDPK4-inhibited, **(C)** PKG-inhibited and proteasome-inhibited microgametocytes, treated with DMSO, 1294, ML10 and bortezomib, respectively. **(A)** Pore-formation and possible echinocytosis of the host cell membrane was found to occur during egress of untreated microgametocytes, which ejected from a single spindle pole of the parasite. Nuclear segregation was found to occur perpendicularly to the mitotic spindle. **(B)** CPDK4 inhibition prevented MTOC transformation and full rounding-up with three distinct egress phenotypes: 1) stress-induced egress prior to activation, 2) no egress and 3) incomplete egress. **(C)** Inhibition of PKG prevented rounding-up and any microtubule polymerisation, with 3 distinct egress phenotypes: 1) no egress, 2) full egress and 3) incomplete egress. **(D)** Proteasome inhibition resulted in abhorrent MTOC division and some nuclear segregation, with few truncated axonemes nucleating from one pole of the transformed parasite. Rounding-up of proteasome-inhibited parasites was observed.

CDPK4 inhibition by 1294 [32, 38] prevented morphological transformation from falciform to round (**Fig 4A** and **4G**), DNA replication (**Fig 4F**) and microtubule polymerisation (**Fig 4E**) during microgametogenesis (**S18–S22 Videos**). On detailed inspection, 1294-treated parasites, failed to reach the maximum level of cell circularity (**Fig 4G**) and SiR-tubulin intensity (**Fig 4A** and **4E**), indicating a role of CDPK4 in microgametocyte rounding as well as cytoskeletal rearrangement during microgametogenesis. Of the population of 1294-treated microgametocytes, 50% were able to fully egress compared to the 53% of untreated microgametocytes which egressed from the host erythrocyte (**Fig 4H**). Whilst these figures are very similar, many 1294-treated microgametocytes were observed to have egressed from the onset of activation, a probable stress-response of CDPK4 inhibition (**Fig 4A**). A few instances of the incomplete egress phenotype in which falciform parasites partially emerged from the host erythrocyte were also observed, with 15% of 1294-treated parasites (n = 3) demonstrating this phenotype (**Fig 4H**). Although the incomplete egress phenotype was not abundant, no instances were seen in in untreated controls (**Fig 4H**). 3D data revealed the positioning of the MTOC which failed to transform to eight basal bodies (**Fig 4A** and **S19 Video**). This suggests that CDPK4 plays an early role in DNA replication, microtubule polymerisation, rounding up and host erythrocyte egress during microgametogenesis. This phenotype is consistent with published findings on 1294-treatment of *P*. *falciparum* [42] and *P*. *berghei* [32] gametocytes, with the exception that our morphological rounding phenotype is not observed in *P*. *berghei* gametocytes which are already round prior to activation. The deduced overall cellular phenotype of CDPK4 inhibition is summarised in **Fig 5B**.

PKG inhibition by ML10 [39] was observed next, clearly demonstrating arrest of microgametocytes before cell-rounding (**Fig 4B** and **4G** and **S23 and S24 Videos**), replication of DNA (**Fig 4F**) and microtubule polymerisation (**Fig 4E**). ML10-treated gametocytes retained a falciform morphology and a level of SiR-tubulin labelling on par with that seen at the onset of gametogenesis activation (**Fig 4D** and **4E**). SiR-tubulin intensity was significantly lower than untreated microgametocytes at 10 minutes post-activation (**Fig 4E**). The host erythrocyte egress phenotype was mixed, as 50% of ML10-treated cells emerged fully, 42% failed (**Fig 4Bii**) and 8% partially emerged (**Fig 4Bi**) from the host erythrocyte (**Fig 4H**). We can deduce from these images that PKG plays a significant role in regulating MTOC transformation, axoneme nucleation and elongation, DNA replication and rounding up exhibited in microgametogenesis, as summarised in **Fig 5C**. These observations match previously reported studies on PKG during microgametogenesis [40, 42] and does so without the laborious steps required using fixed parasite labelling and imaging.

### The microgametocyte proteasome plays a crucial role in microgametogenesis

To extend the cellular dissection of microgametogenesis, we next sought to test the role of the proteosome in cellular reorganisation of the microgametocyte using the drug bortezomib, a proteasome-inhibitor that has not been explored during microgametogenesis. Bortezomib is active against the asexual blood stages of *Plasmodium* [43] and the eukaryote and euryarchaeota proteasomes [44–46]. A recent study on the 20S proteasome of *Sulfolobus acidocaldarius* reported bortezomib arrested cells in the midst of division [47]. Given the role of the proteasome in *S*. *acidocaldarius* cell division and interest in its use as an antimalarial drug target [48] we hypothesised that the proteasome may play a role in cell cycle regulation of microgametogenesis.

Proteasome inhibition by bortezomib resulted in a block of microgametogenesis, preventing nucleation and elongation of eight axonemes (**Fig 4C, 4D and 4E**), DNA replication (**Fig 4F**) and exflagellation (**Fig 4C**). The egress phenotype was mixed with bortezomib treatment, as depicted in **Fig 4H**. We observed inhibition of the *P*. *falciparum* proteasome impacted the transformation of the microgametocyte MTOC, visible as 2 small nodes of SiR-tubulin labelling which remained at one pole of the parasite (**Fig 4Ci** and **S25 Video**). The transformed MTOC was subsequently able to nucleate axonemes from one end of the parasite, but no more than 3 axonemes were formed and growth was truncated (**Fig 4Cii and** 4**Ciii** and **S25–S28 Videos**). This perturbation to axoneme nucleation and elongation resulted in a significant decrease in SiR-tubulin labelling intensity (**Fig 4E**). Additionally, the partial microtubule polymerisation which resulted in the formation of truncated axonemes was reflected in **Fig 4D**, as SiR-tubulin levels increased from ~6 minutes but failed to reach the maximal levels observed in untreated controls. Proteasome-inhibited microgametocytes were, however, able to transform from falciform to round (**Fig 4G**).

Bortezomib treatment also significantly reduced Vybrant DyeCycle Violet labelling intensity, signifying a probable indirect inhibitory effect on DNA replication (**Fig 4F**). Additionally, we observed incomplete transformation of the MTOC which resulted in truncated formation of few axonemes from one pole of developing microgametocytes. Combined these data represent the first time that the cellular role of the proteasome during microgametogenesis has been explored. Our findings, summarised in **Fig 5C**, add further weight to bortezomib’s use as a desirable antimalarial drug candidate that is able to at once inhibit asexual *Plasmodium* parasite replication and sexual stage viability.

## Discussion

To date, detailed observation of *P*. *falciparum* microgametogenesis has mostly centred on fixed parasite imaging, with studies utilising immunofluorescence labelling [23] or electron microscopy [13–15]. Although these studies have been pivotal in developing our current understanding of microgametogenesis cell biology, imaging fixed parasites is limited by the extensive sample preparation steps and fails to resolve the dynamic nature of underlying cellular events. Here, we have developed a live-cell imaging approach that benefits from alternating between brightfield, 2D fluorescence and 3D microscopy imaging through time (4D imaging), to capture the dynamics of *P*. *falciparum* microgametogenesis from activation to exflagellation in fine detail. Utilising widefield microscopy, commercially available labelled target-specific dyes, *in vitro P*. *falciparum* culture and an open-source software for analysis we present a methodological approach that will be readily utilisable for other research groups.

Using a combination of a cell-permeable fluorogenic probe, DNA dye and lectin we can label and observe development of microgametocyte microtubules, DNA and the host erythrocyte membrane, respectively. Whilst live fluorescence imaging is often impeded by phototoxicity, here we have devised a method that maximises the length of time-lapse image acquisition without compromising microgametocyte viability. Our approach permits the acquisition of microgametogenesis in full over two stages: early and late development, capturing spindle formation through to full axoneme development and exflagellation, respectively. Importantly, our imaging approach permits volumetric quantification of 3D data, by both fluorescence and brightfield microscopy, through time which we demonstrate as a powerful tool in defining drug phenotypes. Our approach is consequently applicable to future comparative studies of alternative drug treatment and *P*. *falciparum* transgenic cell lines to wild-type phenotypes.

In line with previous studies on microgametogenesis [12–15], we observed the rapid production of basal bodies from a single MTOC to initiate nucleation and elongation of axonemes, simultaneously to DNA replication and egress. Coupling live fluorescence microscopy to fluorescence intensity and 3D analyses, we observed nuclei to segregate and align perpendicularly to basal bodies in the early stages of microgametogenesis. Whilst synchronous segregation of developing microgametocyte genomes and basal bodies has been reported [12], live fluorescence imaging provides novel insight into the positioning of the newly replicated DNA. Furthermore, we find egress involves pore formation and possible echinocytosis of the host erythrocyte membrane. Although these observations provide novel and preliminary insights into erythrocyte membrane behaviour during microgametogenesis, further characterisation is important to conclusively define these events. Dye influx studies, for example, could be implored to elucidate whether the echinocytosis-like trait observed here involves membrane perforation and if this disruption, additionally to pore-formation, is followed by membrane resealing. Previous studies have reported swelling of the host erythrocyte prior to PVM rupture and vesiculation during *P*. *berghei* microgametogenesis [5]. Here, upon erythrocyte pore formation we observed spindle-directed egress of developing *P*. *falciparum* microgametocytes, such that parasites aligned at the erythrocyte pore by a single spindle pole, prior to ejection. This suggests that there may be forces occurring from a single pole of the microgametocyte that drive egress from the host erythrocyte. Future investigation of pore-forming proteins and their role in this process may also be of relevance.

Additionally, we demonstrate the applicability of our workflow to the study of transmission blocking drug phenotypes. We have used the live imaging framework to elucidate the cellular phenotypes of 1294 and ML10, known inhibitors of microgametogenesis regulators CDPK4 and PKG, respectively. We have also defined the proteasome as a crucial component of microgametogenesis regulation and for the first time, we find bortezomib inhibits the proteasome during this developmental process. Bortezomib may therefore have applications as an inhibitor of *P*. *falciparum* transmission, with future improvements to potency by medicinal chemistry development and *in vivo* mosquito transmission testing. This finding points to the importance of the degradation of misfolded proteins and regulation of functional protein abundance in permitting transmission of the *Plasmodium* microgametocytes. Our approach generates reproducible and consistent phenotypes to fixed parasite studies whilst, critically, not requiring complex fixation or labelling steps.

Although our approach evades the requirement for complex fixation, staining or transgenic parasite generation steps currently required for imaging microgametogenesis, we believe that further optimisation may extend the use of our live imaging workflow. For example, whilst we found alternating acquisition between fluorescence and brightfield enabled prolonged acquisition of the early developmental stages of microgametogenesis, fluorescent timelapses were intermittently captured at a maximum of 10-frames. Capturing microgametogenesis in its entirety using fluorescence imaging should undoubtedly be prioritised as the method is further developed. Crucially, this improvement to the workflow would enhance the applicability of the framework to high-throughput campaigns, such as those focussed on the elucidation of the phenotypes of transmission-blocking drug candidates. It is additionally important to highlight that live-imaging of exflagellation was restricted to 2D, given the dynamic nature of emerging microgametes. Although alignment of microgametes can be captured in 3D upon loss of motion, this requires immediate acquisition upon loss of parasite viability to evade capturing artifacts which may arise as a result of cell death, such as morphological changes and reduction in live-cell dye staining. Overcoming many of the existing limitations to microgametogenesis imaging, this live-cell approach has demonstrated the ability to resolve microtubule dynamics, egress and DNA replication events with improved spatiotemporal resolution. Given the accessibility to the wider malaria research community, we hope the protocol lays the groundwork for further development and optimisation. As drug and insecticide resistance has threatened existing antimalarial treatment strategies, there is an urgent need for novel transmission-blocking antimalarials that can be used singularly or in combination with schizonticides, killing asexual stages. Developing a full understanding of the mode of action of antimalarial drug candidates maximises the likelihood of clinical safety and future administration. Our imaging approach permits deeper understanding of this remarkable cell biology process, capturing real-time development with fluorescence which may otherwise be missed with fixed or brightfield imaging. The data depicted here promises to unveil novel insights into *P*. *falciparum* microgametogenesis for cell biology and drug study, but the protocol is not limited to this. Cultivation of *in vitro Plasmodium* cultures, at any stage, permits the live microscopy of the breadth of malaria parasite development, from macrogametogenesis and asexual blood stage development to liver stages. Additional markers for intracellular organelles, parasite membranes and sex-specific proteins, with both wild-type and transgenic lines, will now be a priority for exploring so that we can shed further light on this ancient but deadly single-celled parasite.

## Materials and methods

### *In vitro* culture of *Plasmodium falciparum*

*P*. *falciparum* NF54 strain parasites were cultured as previously described [36]. Asexual parasite cultures were maintained between 0.75–5% parasitaemia and 4% haematocrit using human erythrocytes (NHS National Blood Service). Erythrocytes were supplemented with 3 units/ml heparin (Sigma-Aldrich). Parasites were grown in asexual parasite culture medium (RPMI 1640 with 25 mM HEPES (Life Technologies) supplemented with 50 μg/ml hypoxanthine (Sigma), 0.3 g/l L-glutamine (Sigma) and 10% human serum (Interstate Blood-Bank)). Gametocyte cultures were induced from asexual parasite cultures at 3% asexual parasitaemia and 4% haematocrit. Gametocyte culture media (RPMI 1640 with 25 mM HEPES supplemented with 150 μg/ml L-glutamine, 2.78 mg/ml sodium bicarbonate, 2 mg/ml D-glucose, 50 μg/ml hypoxanthine, 5% human serum and 5% AlbuMAX-II (Gibco)) was replaced daily until reaching maturity at day 14-post induction. All cultures were maintained at 37°C under 3% O_2_/5% CO_2_/93% N_2_ (BOC, UK).

Upon reaching maturity, gametocyte viability was determined by measuring the rate of exflagellation relative to erythrocyte density. Gametocyte culture was treated with ookinete media (RPMI 1640 supplemented with 2 g/l sodium bicarbonate, 50 mg/l hypoxanthine and 100 mM Xanthurenic Acid (XA) (Sigma-Aldrich), pH adjusted to 7.4) to activate gametogenesis. Exflagellation events and erythrocyte density was counted using a haemocytometer (VWR) and Nikon Leica DC500 microscope.

### Labelling and treating live *P*. *falciparum* Gametocytes

For live-cell fluorescence imaging, samples of mature gametocyte culture (> 0.3% exflagellation) were labelled with 500 nM SiR-tubulin (Spirochrome) for 3 hours at 37°C. Samples were additionally labelled with 5 μg/ml wheat germ agglutinin (WGA) conjugated to AlexaFluor488 (Invitrogen) and 500 nM Vybrant DyeCycle Violet for 30 minutes at 37°C. Labelled samples were protected from light and strictly maintained at 37°C, to prevent premature activation, until imaging.

Samples were treated with 10 μM 1294 [49],10 μM ML10 [39], 25 μM Bortezomib or DMSO and normalised to 0.25% DMSO for 3 hours at 37°C, before imaging.

### Imaging live microgametogenesis by Widefield microscopy

Wells of an Ibidi 8-well μ-Slide were pre-treated with 140 μl ookinete medium and slides were pre-positioned on the microscope stage. To activate gametogenesis, 6 μL of the labelled gametocytes, equating to ~30 million total erythrocytes, was added directly to ookinete media-treated wells at room temperature (21°C). Samples were imaged with a Prime 95B sCMOS camera (photometrics) on a Nikon Ti2-E widefield microscope using x 100 Plan Apo 1.4 numerical aperture (NA) oil objective with NIS Elements v4.20 software. SiR-Tubulin, WGA-488 and Vybrant DyeCycle Violet labelling were imaged with a Cy5, GFP and DAPI filter set. The triggered multi-wavelength LED and static quad band filter cube was used in acquisition through Z and between wavelengths.

Early stages of microgametogenesis (0–10 minutes) were acquired as 3D datasets through time at ‘no delay’ and with 34 ms exposure time. Z-stacks were acquired at 0.2 μm steps from above and below the cell with a Piezo driven stage. Acquisition was alternated between brightfield and fluorescence to minimise phototoxicity and subsequently prolong parasite viability. Exflagellation was captured as 2D (single z-slice) datasets, acquiring with no delay between frames.

### Image analysis

The open-source bioimage analysis software Icy [50] was used to analyse all time-lapse datasets. All 3D datasets are depicted here as 2D maximum intensity projections. Early developmental stages of microgametogenesis, acquired through t and z, were deconvolved using a custom-made Protocol in Icy. The Protocol is attached as **S1 File**. Within the Protocol, a Sequence File Batch loop locates the 3D+t files from which a channel of interest is extracted. The selected channel of each time frame is processed as an individual 3D stack. The metadata of each stack is read and used as an input for the EpiDEMIC deconvolution bloc (Epifluorescence Deconvolution MICroscopy), a blind (i.e. without Point Spread Function (PSF) knowledge) deconvolution method for widefield fluorescence microscopy 3D data [51]. All timelapses were deconvolved over 50 iterations and 2 loops.

All 2D time-lapse data and 3D data depicted as 2D maximum intensity projections or 3D sectioned views were created using Icy. To quantify SiR-tubulin and Vybrant DyeCycle Violet intensity, egress phenotypes and cell circularity, raw 3D data was converted to 2D maximum intensity projections in NIS Elements v4.20 prior to analysis. To calculate SiR-Tubulin volume, staining intensity was multiplied by the 3D volume of SiR-Tubulin stained content, obtained using the 3D Analysis tool in Icy [50]. Egress phenotypes were quantified by manual observation. To measure the circularity and labelling intensity of individual cells, each cell was defined as a custom region of interest. The SiR-tubulin and Vybrant DyeCycle Violet labelling intensity of each individual cell was quantified using the time-measurement function in NIS Elements v4.20 and is reported in arbitrary units. The circularity of each cell was measured using the Automated Measurement feature in NIS Elements v4.20. Circularity is reported here as shape measure values from 0–1 derived from the area and perimeter of each cell, with higher values representing shapes of increasing circularity and circles being characterised as a value of 1.

To measure the angle of DNA segregation relative to basal body tetrads, frames corresponding to appropriate developmental stages (spindle, newly nucleated axonemes, developed axonemes) were selected from 4D timelapses (3D data acquired through time). 3D frames were oriented for optimal angle measurement and the maximum intensity projection was obtained in Icy [50]. Segregated DNA and basal body tetrads were marked and the ‘angle helper’ tool was used to measure the angle between points.

All graphical and statistical data was analysed with GraphPad Prism version 8.0.

## Supporting information

S1 Fig Individual channels of 3-colour *P*. *falciparum* microgametogenesis time-lapse data. Individual channels of **(A)** tubulin dynamics from **Fig 2B**, **(B)** host erythrocyte egress from **Fig 2D, (C-D)** additional egress data and **(E)** exflagellation from **Fig 3C**. Merged channels, microtubules (SiR-Tubulin), host erythrocyte membrane (WGA) and parasite nuclei (Vybrant DyeCycle Violet) of 2D maximum intensity projection data is shown. Time is depicted as minutes and seconds (mm:ss) in **A-D** and minutes, seconds and milliseconds (mm:ss:ms) in **E**. Scale bars = 10 μm.
(TIF)

S2 Fig Early emergence of *P*. *falciparum* microgametes. The merged image, microtubules (SiR-Tubulin), host erythrocyte membrane (WGA) and parasite nuclei (Vybrant DyeCycle Violet) of microgametogenesis in the early stages of exflagellation. Images represent stills derived from timelapses, portrayed as 2D maximum intensity projection of 3D data. See **S7 Video** for the corresponding time-lapse. Time is depicted as minutes, seconds and milliseconds (mm:ss:ms). Scale bars = 10 μm.
(TIF)

S3 Fig Individual channels of CDPK4, PKG and proteasome-inhibited parasites. Individual channels depicting the phenotypes of **(A)** CDPK4, **(B)** PKG and **(C)** proteasome-inhibition by 1294, ML10 and bortezomib, respectively. 2D maximum intensity projection images are depicted and accompany **Fig 4A–4C**. Merged channels, microtubules (SiR-Tubulin), host erythrocyte membrane (WGA) and parasite nuclei (Vybrant DyeCycle Violet) are shown. Time is depicted as minutes and seconds (mm:ss). Scale bars = 10 μm.
(TIF)

S1 File Batch Deconvolution Protocol.protocol. Description: Custom-made protocol file comprised of processing steps, termed Blocs, for batch deconvolution of 3D time-lapse data in Icy.
(PROTOCOL)

S1 Video Description: SiR-tubulin (magenta) stained developing microgametocytes during microgametogenesis. The microtubule organising centre (MTOC) within the falciform gametocyte is shown to transform, a spindle forms and axonemes are nucleated and elongated from basal bodies. Accompaniment for **Fig 2A**.
(AVI)

S2 Video Description: 3D sectioned view of early developmental stages of microgametogenesis. SiR-Tubulin (magenta), WGA-488 (green) and Vybrant DyeCycle Violet (blue). The 2D maximum intensity projection (MIP) of 3D frames is shown. Accompaniment for **Figs 2B** and **S1A**.
(AVI)

S3 Video Description: 3D sectioned view of early microgametogenesis and egress. SiR-Tubulin (magenta), WGA-488 (green) and Vybrant DyeCycle Violet (blue). The 2D maximum intensity projection (MIP) of 3D frames is shown. Accompaniment for **Figs 2C** and **S1B**.
(AVI)

S4 Video Description: Maximum intensity projection of early microgametogenesis, from mitotic spindle to lengthened axonemes. SiR-Tubulin (magenta), WGA-488 (green) and Vybrant DyeCycle Violet (blue). The 2D maximum intensity projection (MIP) of 3D frames is shown.
(AVI)

S5 Video Description: 3D sectioned view of early microgametogenesis and egress. SiR-Tubulin (magenta), WGA-488 (green) and Vybrant DyeCycle Violet (blue). The 2D maximum intensity projection (MIP) of 3D frames is also shown. Accompaniment for **S1C Fig**.
(AVI)

S6 Video Description: 3D sectioned view of early microgametogenesis and egress. SiR-Tubulin (magenta), WGA-488 (green) and Vybrant DyeCycle Violet (blue). The 2D maximum intensity projection (MIP) of 3D frames is also shown. Accompaniment for **S1D Fig**.
(AVI)

S7 Video Description: 2D maximum intensity projection (MIP) of 3D time-lapse data depicting early microgamete emergence. **(A)** SiR-Tubulin (magenta), WGA-488 (green) and Vybrant DyeCycle Violet (blue) (accompaniment for **S2 Fig**) and corresponding **(B)** brightfield frames.
(AVI)

S8 Video Description: 2D (single Z-slice) time-lapse of exflagellation, brightfield information depicted. Accompaniment for **Fig 3A**.
(AVI)

S9 Video Description: 2D (single Z-slice) time-lapse of exflagellation, brightfield information depicted. (AVI)

S10 Video Description: 2D (single Z-slice) time-lapse of exflagellation of a parasite stained with SiR-tubulin (magenta). Accompaniment for **Fig 3B**.
(AVI)

S11 Video Description: 2D (single Z-slice) time-lapse of exflagellation a parasite stained with SiR-tubulin (magenta).
(AVI)

S12 Video Description: 2D (single Z-slice) time-lapse of exflagellation of a parasite stained with SiR-tubulin (magenta).
(AVI)

S13 Video Description: 2D (single Z-slice) time-lapse of exflagellation of a parasite stained with SiR-tubulin (magenta).
(AVI)

S14 Video Description: 2D (single Z-slice) time-lapse of exflagellation of a parasite stained with SiR-tubulin (magenta).
(AVI)

S15 Video Description: 2D (single Z-slice) time-lapse of exflagellation of a parasite stained with SiR-tubulin (magenta), WGA-488 (green) and Vybrant DyeCycle Violet (blue). Accompaniment for **Figs 3C** and **S1E**.
(AVI)

S16 Video Description: 2D (single Z-slice) time-lapse of exflagellation of a parasite stained with SiR-tubulin (magenta), WGA-488 (green) and Vybrant DyeCycle Violet (blue).
(AVI)

S17 Video Description: Rotated view of 3D view of an exflagellating parasite stained with SiR-tubulin (magenta), WGA-488 (green) and Vybrant DyeCycle Violet (blue). A single time-frame is shown. Accompaniment for **Fig 3E**.
(AVI)

S18 Video Description: 2D maximum intensity projection (MIP) of 3D time-lapse data depicting 1294-treatment phenotype. SiR-Tubulin (magenta), WGA-488 (green) and Vybrant DyeCycle Violet (blue).
(AVI)

S19 Video Description: 3D sectioned view of 1294-treatment phenotype. SiR-Tubulin (magenta), WGA-488 (green) and Vybrant DyeCycle Violet (blue). The 2D maximum intensity projection (MIP) of 3D frames is shown. Accompaniment for **Fig 4A**.
(AVI)

S20 Video Description: 2D maximum intensity projection (MIP) of 3D time-lapse data depicting 1294-treatment phenotype. SiR-Tubulin (magenta), WGA-488 (green) and Vybrant DyeCycle Violet (blue).
(AVI)

S21 Video Description: 2D maximum intensity projection (MIP) of 3D time-lapse data depicting 1294-treatment phenotype. SiR-Tubulin (magenta), WGA-488 (green) and Vybrant DyeCycle Violet (blue).
(AVI)

S22 Video Description: 2D maximum intensity projection (MIP) of 3D time-lapse data depicting 1294-treatment phenotype. SiR-Tubulin (magenta), WGA-488 (green) and Vybrant DyeCycle Violet (blue).
(AVI)

S23 Video Description: 3D sectioned view of ML10-treatment phenotype. SiR-Tubulin (magenta), WGA-488 (green) and Vybrant DyeCycle Violet (blue). The 2D maximum intensity projection (MIP) of 3D frames is shown. Accompaniment for **Fig 4B**.
(AVI)

S24 Video Description: 3D sectioned view of ML10-treatment phenotype. SiR-Tubulin (magenta), WGA-488 (green) and Vybrant DyeCycle Violet (blue). The 2D maximum intensity projection (MIP) of 3D frames is shown. Accompaniment for **Fig 4B**.
(AVI)

S25 Video Description: 3D sectioned view of Bortezomib-treatment phenotype. SiR-Tubulin (magenta), WGA-488 (green) and Vybrant DyeCycle Violet (blue). The 2D maximum intensity projection (MIP) of 3D frames is shown. Accompaniment for **Fig 4C**.
(AVI)

S26 Video Description: 2D maximum intensity projection (MIP) of 3D time-lapse data depicting Bortezomib-treatment phenotype. SiR-Tubulin (magenta), WGA-488 (green) and Vybrant DyeCycle Violet (blue).
(AVI)

S27 Video Description: 2D maximum intensity projection (MIP) of 3D time-lapse data depicting Bortezomib-treatment phenotype. Accompaniment for **Fig 4C**.
(AVI)

S28 Video Description: 2D maximum intensity projection (MIP) of 3D time-lapse data depicting Bortezomib-treatment phenotype. Accompaniment for **Fig 4C**.
(AVI)

S1 Data Description: All numerical data used to obtain graphical Figs, listed as corresponding Fig names. (XLSX)
