# Supplementary figures and images for: Live-cell fluorescence imaging of microgametogenesis in the human malaria parasite Plasmodium falciparum
Source: PLoS Pathog. 2022 Feb 7;18(2):e1010276. doi: 10.1371/journal.ppat.1010276 (PMC8853644; doi:10.1371/journal.ppat.1010276)

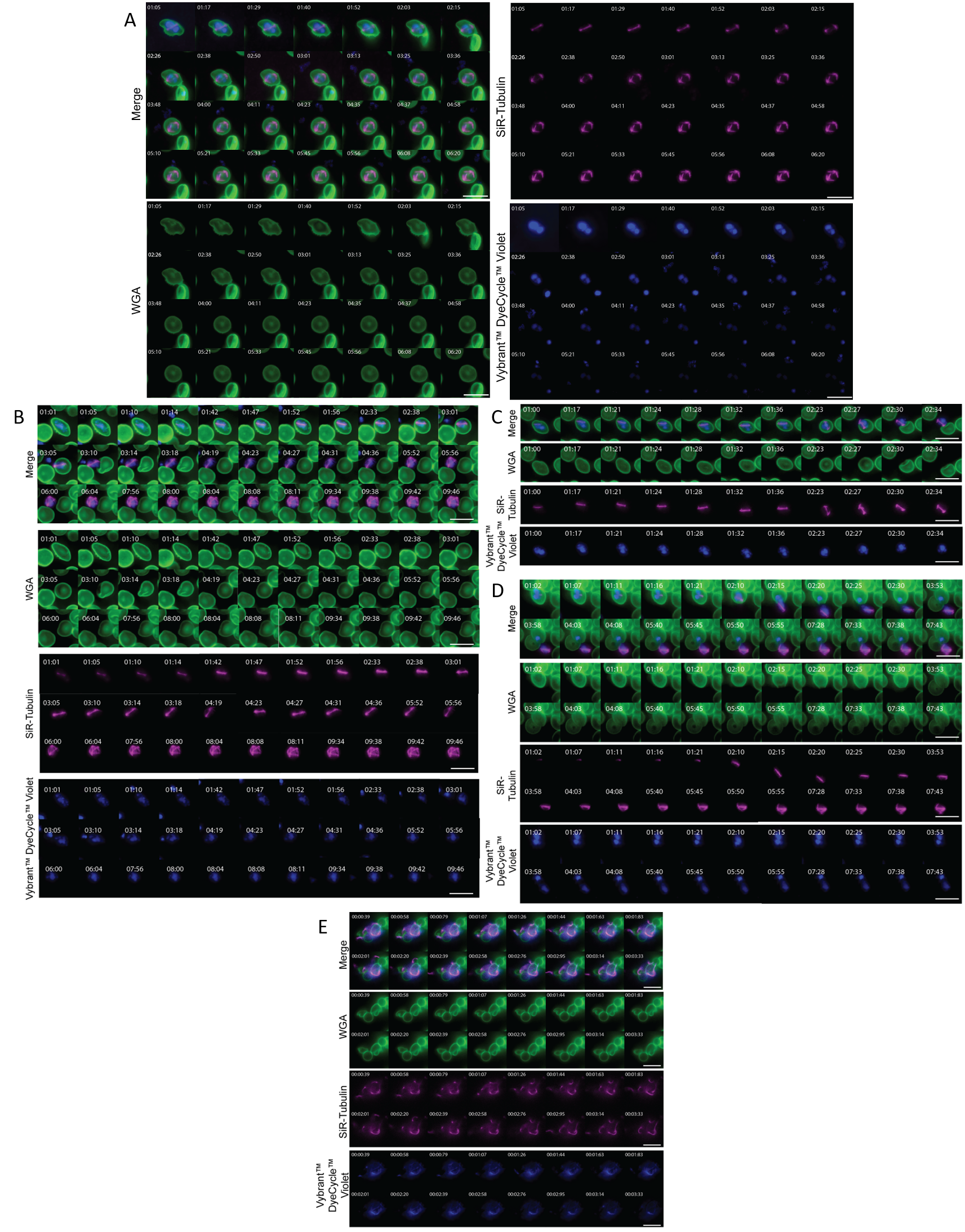

Supplement: S1 Fig — Individual channels of (A) tubulin dynamics from Fig 2B, (B) host erythrocyte egress from Fig 2D, (C-D) additional egress data and (E) exflagellation from Fig 3C. Merged channels, microtubules (SiR-Tubulin), host erythrocyte membrane (WGA) and parasite nuclei (Vybrant DyeCycle Violet) of 2D maximum intensity projection data is shown. Time is depicted as minutes and seconds (mm:ss) in A-D and minutes, seconds and milliseconds (mm:ss:ms) in E. Scale bars = 10 μm. (TIF) [file ppat.1010276.s001.tif]

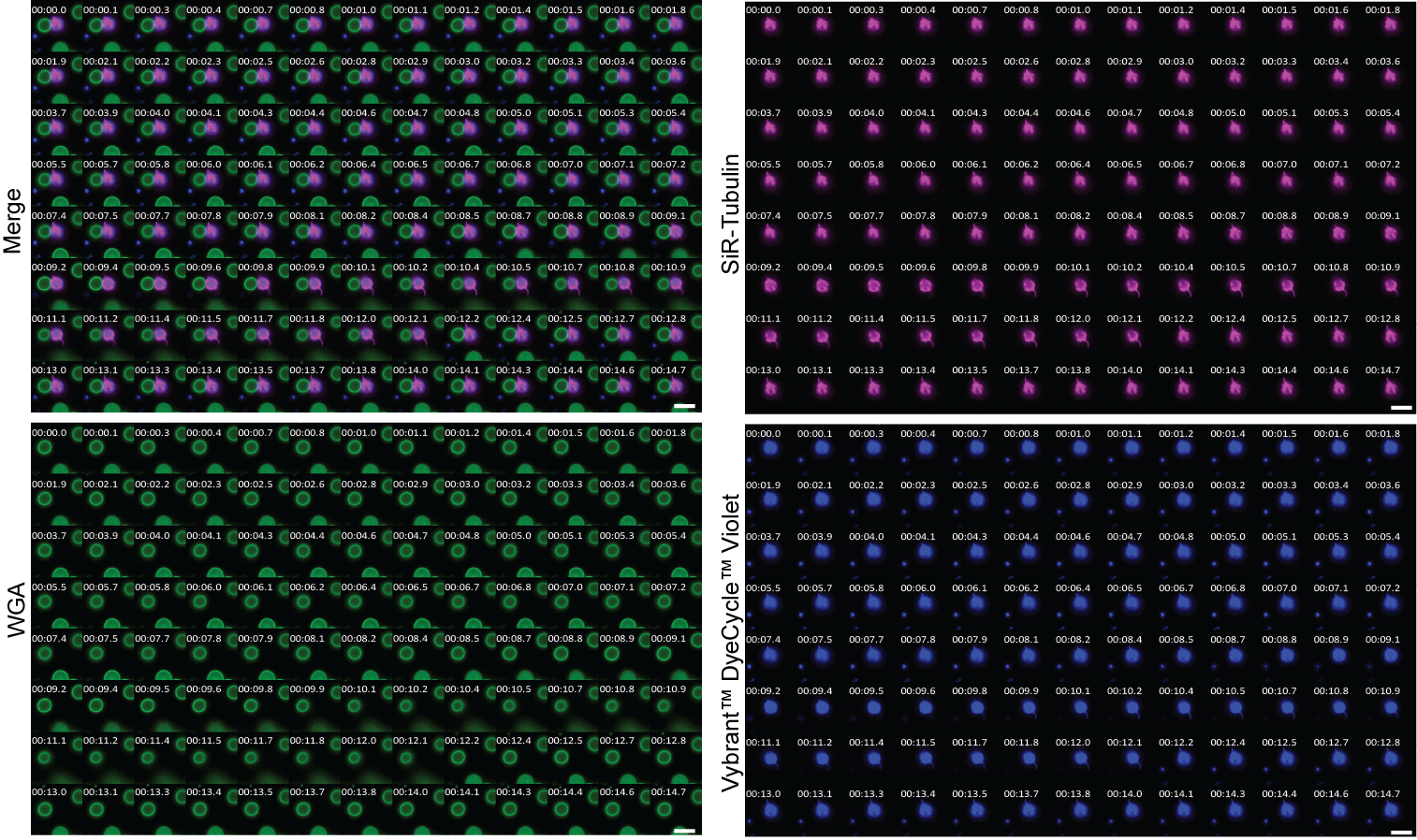

Supplement: S2 Fig — The merged image, microtubules (SiR-Tubulin), host erythrocyte membrane (WGA) and parasite nuclei (Vybrant DyeCycle Violet) of microgametogenesis in the early stages of exflagellation. Images represent stills derived from timelapses, portrayed as 2D maximum intensity projection of 3D data. See S7 Video for the corresponding time-lapse. Time is depicted as minutes, seconds and milliseconds (mm:ss:ms). Scale bars = 10 μm. (TIF) [file ppat.1010276.s002.tif]

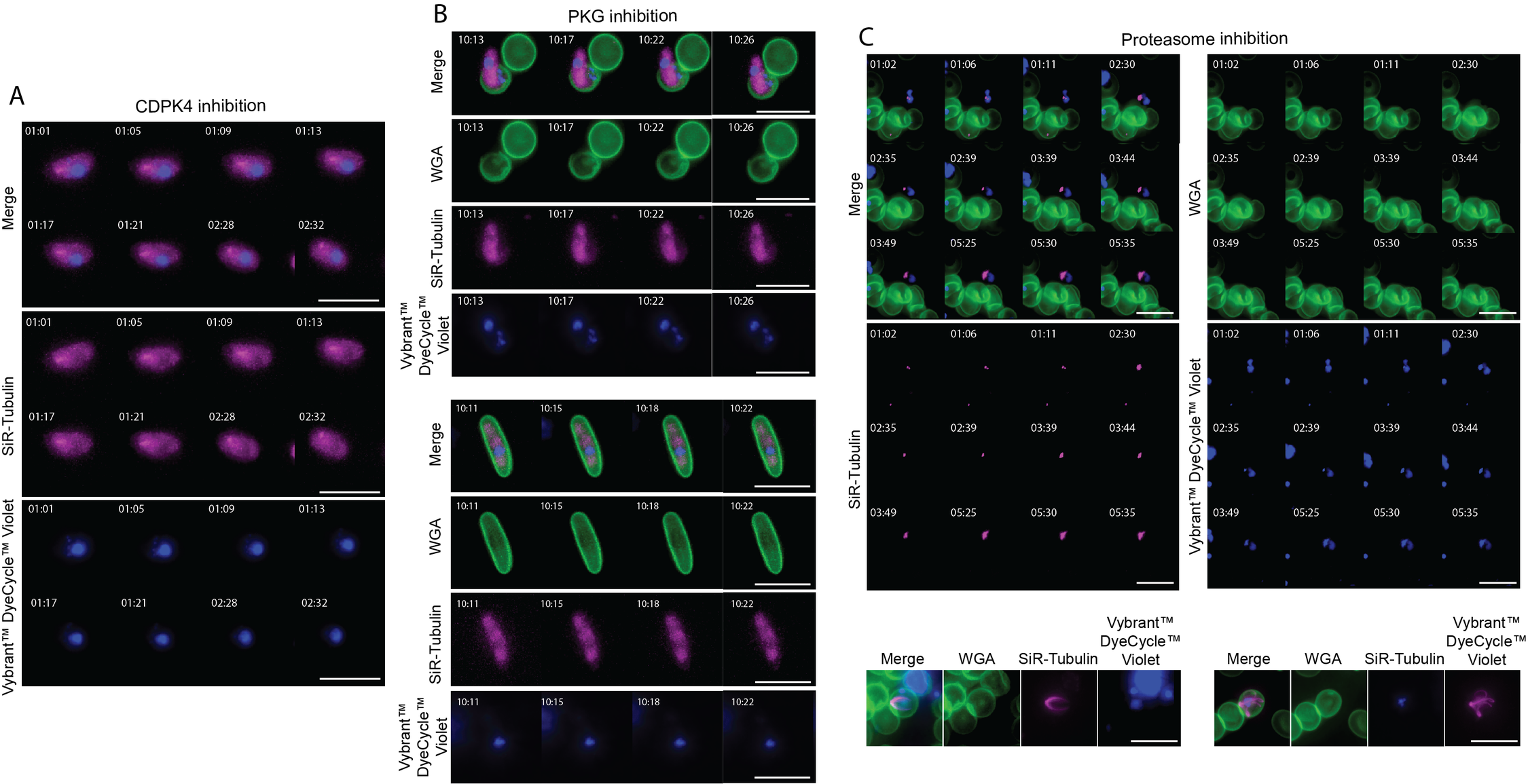

Supplement: S3 Fig — Individual channels depicting the phenotypes of (A) CDPK4, (B) PKG and (C) proteasome-inhibition by 1294, ML10 and bortezomib, respectively. 2D maximum intensity projection images are depicted and accompany Fig 4A–4C. Merged channels, microtubules (SiR-Tubulin), host erythrocyte membrane (WGA) and parasite nuclei (Vybrant DyeCycle Violet) are shown. Time is depicted as minutes and seconds (mm:ss). Scale bars = 10 μm. (TIF) [file ppat.1010276.s003.tif]
